# High-resolution 3D visualization of human hearts with emphases on the cardiac conduction system components—a new platform for medical education, mix/virtual reality, computational simulation

**DOI:** 10.3389/fmed.2025.1507005

**Published:** 2025-02-18

**Authors:** Weixuan Chen, Marcin Kuniewicz, Abimbola J. Aminu, Irem Karaesmen, Neal Duong, Klaudia Proniewska, Peter van Dam, Tinen L. Iles, Mateusz K. Hołda, Jerzy Walocha, Paul A. Iaizzo, Michael A. Colman, Halina Dobrzynski, Andrew J. Atkinson

**Affiliations:** ^1^Division of Cardiovascular Sciences, School of Medical Sciences, University of Manchester, Manchester, United Kingdom; ^2^Jagiellonian University Medical College, Krakow, Poland; ^3^The Visible Heart^®^ Laboratories and the Institute for Engineering in Medicine, University of Minnesota, St. Paul, MN, United States; ^4^Department of Cardiology, University Medical Centre Utrecht, Utrecht, Netherlands; ^5^Department of Surgery, University of Minnesota, St. Paul, MN, United States; ^6^School of Biomedical Sciences, Faculty of Biological Sciences, University of Leeds, Leeds, United Kingdom

**Keywords:** cardiac conduction system, micro-CT, computational simulation, 3D printing, reconstruction, myocardial infarction, virtual reality, 3D visualization

## Abstract

**Introduction:**

High-resolution digitized cardiac anatomical data sets are in huge demand in clinical, basic research and computational settings. They can be leveraged to evaluate intricate anatomical and structural changes in disease pathology, such as myocardial infarction (MI), which is one of the most common causes of heart failure and death. Advancements in high-resolution imaging and anatomical techniques in this field and our laboratory have led to vast improvements in understanding cardiovascular anatomy, especially the cardiac conduction system (CCS) responsible for the electricity of the heart, in healthy/aged/obese post-mortem human hearts. However, the digitized anatomy of the electrical system of the heart within MI hearts remains unexplored.

**Methods:**

Five post-mortem non-MI and MI human hearts were obtained by the Visible Heart^®^ Laboratories via LifeSource, Minneapolis, MN, United States (with appropriate ethics and consent): specimens were then transported to Manchester University with an material transfer agreement in place and stored under the HTA 2004, UK. After performing contrast-enhanced micro-CT, a visualization tool (namely Amira) was used for 3D high-resolution anatomical visualizations and reconstruction. Various cardiovascular structures were segmented based on the attenuation difference of micro-CT scans and tissue traceability. The relationship between the CCS and surrounding tissues in MI and non-MI human hearts was obtained. 3D anatomical models were further explored for their use in computational simulations, 3D printing and mix/virtual reality visualization.

**Results:**

3D segmented cardiovascular structures in the MI hearts elicited diverse macro-/micro- anatomical changes. The key findings are thickened valve leaflets, formation of new coronary arteries, increased or reduced thicknesses of pectinate and papillary muscles and Purkinje fibers, thinner left bundle branches, sinoatrial nodal atrophy, atrioventricular conduction axis fragmentation, and increased epicardial fat in some hearts. The propagation of the excitation impulses can be simulated, and 3D printing can be utilized from the reconstructed and segmented structures.

**Discussion:**

High-resolution digitized cardiac anatomical datasets offer exciting new tools for medical education, clinical applications, and computational simulation.

## 1 Introduction

Myocardial infarction (MI) and coronary artery disease (CAD) are the two most common causes of heart failure and death. Currently, there are over 200 million CAD patients worldwide, and 3.8% of individuals under 60 years of age have associated MI (1). MI is commonly caused by obstruction of coronary arteries and is defined as myocardial cell death due to prolonged myocardial ischemia. Known risk factors for MI/CAD include obesity, high blood pressure, high cholesterol levels, smoking, and/or diabetes. An MI may also lead to either dilated or hypertrophic cardiomyopathy. In dilated cardiomyopathy, the ventricular walls are thinner, and the chambers become dilated (2). In hypertrophic cardiomyopathy, the walls are thickened, the myocardium becomes stiff, and the filling volumes are lowered (3).

In traditional medical education, students tend to use textbooks and cadavers to learn anatomy, however, they do not provide the learner with any depth perception and/or detailed anatomy of small structures. The 3D imaging software such as Amira, virtual reality (VR), and 3D printing give students a more comprehensive and accurate understanding of the complex human heart anatomy (4–6). The use of the 3D approach can also be expanded to clinical settings, computational simulation of various arrythmias and medical device development.

Furthermore, high-resolution 3D anatomy approaches can be used to visualize real anatomy and may be applied to more precise surgical planning, diagnosis, and/or basic and translational scientific research (7, 8). For example, 3D-printed models can replicate the complex normal and diseased human anatomy and provide pre-operational practices for surgeons and precise measurements for more suitable stents in treating aortic diseases (9, 10).

The cardiac conduction system (CCS) can be differentiated from the surrounding working myocardium using high-resolution imaging techniques. In the previously published paper from our group, the normal CCS in one healthy human heart was shown by analyzing micro-computed tomography (micro-CT) scans (11). Then, in our proceeding paper, the CCS from one aged heart and one obese heart were compared with one healthy human heart using the same micro-CT technique, to show the structural differences in aged and obese hearts (12). In this study, we present the expansions of our previous studies and the utilization of this technique to study the CCS anatomy further. Therefore, the main purpose of this research is to illustrate the complex 3D CCS anatomy in four MI *ex-vivo* human hearts and compare to one non-MI heart and offer new tools to study the complexity of the CCS and other cardiac structures, vessels and valves to be applied to basic and clinical and computational settings.

## 2 Materials and methods

### 2.1 Human sample details and ethical approval

Five *ex-vivo* post-mortem human hearts were obtained from the Visible Heart^®^ Laboratories (VHL) at the University of Minnesota, United States, via LifeSource, Minneapolis, MN, USA, under their appropriate local ethical rules. Detailed de-identified patient information is provided in Table 1 and is available from the VHL website.^1^ The hearts were perfusion-fixed with 10% formalin, as previously described in Stephenson et al. (11), and transported to the University of Manchester following a Material Transfer Agreement and stored under the HTA 2004, UK.

**TABLE 1:** Patient profile of the human hearts involved and color code of segmented structures.

Heart number	Sex	Age	BMI	Body weight (kg)	Body height (cm)	Cardiovascular medical history and cause of death	Structures segmented
0059 Non-MI	F	54	20.7	53	160	Healthy heart Cerebrovascular accident	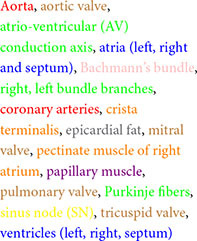
0301 MI	M	43	22.1	72	180	Hypertension, MI (2 months prior to death), cardiomyopathy, severe CAD, stenting Electronic pacemaker Conduction block (P wave missing), QRS wide, QT prolonged, ST changes, ischemia, anoxia	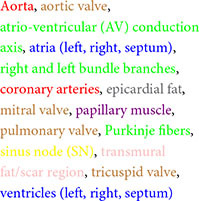
0244 MI	M	50	31.1	98	178	Hypertension, MI (15 years prior to death), serve CAD, stenting HR 145 bpm PR prolonged > 120ms, QRS wide, ST-T changes, ischemia Conduction defect, VF, anoxia Cardiac arrest	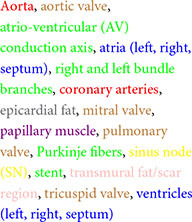
0220 MI	M	51	32.7	98	170	EF 20%, systolic function reduced, MI, mitral valve leaflet thickening, regurgitation Severe CAD, stenting, aortic aneurysm HR 128 bpm, MI, cardiac arrest	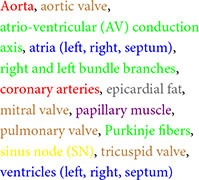
0350 MI	M	71	31.2	99	178	Hypertension, MI (15 years prior to death), stenting HR 56-124 bpm, PR missing or normal, QT normal to prolonged > 400 ms T wave abnormal	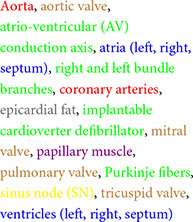

### 2.2 Micro-CT scanning workflow

#### 2.2.1 Heart tissue preparation and micro-CT scanning

The hearts were briefly washed in distilled water to remove excess formalin and were immersed in a 7.5% aqueous iodine-potassium iodide (I_2_KI) contrast medium for 14 days. The I_2_KI was changed after day 7. After diffusion staining, the hearts were rinsed in distilled water for 24 hours to rinse off the excessive contrast medium. 2% agarose solution or plastic bags were used to immobilize the hearts and preserve the cardiac structure within a humid plastic scan container as previously described in Stephenson et al. and Stephenson and Atkinson et al. (11, 13).

Micro-CT scanning was performed using Nikon Metris XTEK 320kV Custom Bay, Nikon XTEK XTH 225 kV and the High Flux XTEK bay systems at the Henry Moseley X-Ray Imaging Facility, University of Manchester. 360° scans were performed, and 85–160 kV X-ray energies were used during scanning. The scanned data was collected from 2000 to 3142 projections with two frame averaging, and a 1mm copper filter was used during the scanning. The filtered back projection was used for the reconstruction of the micro-CT images from tomographic data, and the data was exported as a .tiff image stack. The voxel size obtained for each heart is shown in Supplementary Table S1.

#### 2.2.2 Micro-CT 3D reconstructions

The micro-CT scans in the .tiff image stack format were uploaded to Amira 6.5 (Thermo Fisher Scientific) and all processing, segmentation and 3D reconstruction was performed using this software. The “volume rendering” function was used to visualize the high-resolution scan datasets. The description of detailed tissue preparation and micro-CT scanning can be found in the supplemental methods section.

#### 2.2.3 Ventricular wall thickness and chamber diameter measurements

The right ventricular free wall, ventricular septum, and left ventricular free wall thicknesses, as well as right ventricle (RV) and left ventricle (LV) chamber diameters, were measured in Amira 6.5. The measurements are listed in Supplementary Table S1.

The “slice” function in Amira was used to freely select the appropriate 2D planes for measurement analysis. Three short-axis/transverse planes were selected. The base plane was determined by the plane perpendicular to the ventricular septum and just inferior to the tricuspid and mitral valve annulus. The apex plane was determined by the plane parallel to the base plane and just superior to the apex of the heart where the two ventricles are still visible. The mid plane was determined by the mid-point of the base planes and apex plane and is parallel to two planes. Three long-axis/coronal planes were also selected. The ventral/anterior plane was determined by visualizing the anterior portion of the heart where two ventricles can be seen and just separated by the ventricular septum. The posterior/dorsal plane was established in the same manner but from the posterior aspect of the heart. The mid plane was determined as the midpoint between the anterior and posterior planes and parallel to the two planes.

The “3D length” under the “measurement” function within Amira was used to measure ventricular wall thickness. Three right ventricular free wall, three ventricular septum and three left ventricular free wall measurements were taken in each plane.

The base and the mid plane of short-axis/transverse orientation and the mid plane of long-axis/coronal orientation were used for chamber diameter measurements. The chamber diameter measurements were taken at the widest point of the chambers (for short-axis/transverse orientation) or just inferior to the attachments of tricuspid and mitral valves (for long-axis/coronal orientation).

#### 2.2.4 Segmentations and volume measurements

The specialized segmentation tools within Amira were used to segment the structures of interest based on various special characteristics of each tissue, including signal intensity differences, distinct anatomical features, and serial slices traceability. After segmentation, the “generate volume” function within Amira was used to visualize and create a 3D mesh from which the volume of different structures could be obtained. The relative positioning and relationship of structures could also be assessed at this point. 3D meshes were exported in .stl formats for mix/virtual reality and 3D printing. Segmentation data for all structures of interest were exported as 8-bit image label files suitable for use in computational simulation software.

In Figures, Supplementary Figures and Table 1, the colors were assigned for each segmented structure for illustrative and educational purposes. The atria were segmented in sky blue and included the pectinate muscles and surrounding epicardial fat and connective tissue located superior to the mitral and tricuspid valves. In heart 0059, the crista terminalis (CT) was segmented and displayed in orange. It was traced from the most anterior slice where the muscle combined with the superior vena cava (SVC) and segmented serially toward the posterior atrium until the muscle was defined again. The pectinate muscles of the right atrium that originate from the CT were segmented and shown in light orange and Bachmann’s bundle was defined in pink. This bundle/tract was identified by the muscle bundle that can be traced from the anterior of the CT, close to the head of the sinus node (SN), toward the left atrium. The ventricular muscle was segmented and shaded in blue and included all tissue that was located inferior to the attachment of mitral and tricuspid valves. The myocardium, epicardial fat and papillary muscles were all included in these detailed ventricle segmentations. Epicardial fat was segmented and shaded in gray, and it was identified by the attenuation differences from the working myocardium. The transmural fat/scar region (if observed) was segmented and defined in light pink. Such an MI region was identified as a structure located in the myocardium layer but has attenuation differences from the surrounding viable myocardium. The aorta was segmented and colored in red, from the inferior end of the aortic root to about 3 cm superior to the aortic valve. The coronary arteries were identified and noted in red, and they were traced from the coronary ostia at the base of the aortic valves until the vascular tissue could not be identified. Papillary muscles were identified and shaded in purple. They were traced from the cordae tendineae of the mitral and tricuspid valves and segmented to their endocardial attachments. The atrioventricular and semilunar valves were segmented and depicted in brown. The SN was segmented based on the visual attenuation differences of the tissue slices in the intercaval region of the right atrium (RA) close to the CT, and it was segmented and noted in yellow. The atrioventricular conduction axis and Purkinje fibers were serially traced as continuous structures from the distinct penetrating bundle structure in the central fibrous body and were carefully segmented and depicted in green. It has to be noted that we followed the location of AVCA structures according to the recent publication from Anderson et al., who elegantly and precisely described the location of these structures (14). In heart 0244, an implanted stent was found within the right coronary artery. It was segmented and shaded in light green. It was defined by the distinct attenuation differences from the coronary artery wall and had an identified wire shape. In heart 0350, a biventricular implantable cardioverter defibrillator (ICD) was detected with three leads going into the RA, RV, and LV. Due to their metallic compositions, these leads have unique attenuation differences from the rest of the anatomical structures.

#### 2.2.5 Video-making

Amira software was used and Supplementary Videos S1–S5 were made before the segmentation. The “video orbit” function was used. Supplementary Videos S6–S10 were made after the segmentation was performed.

A software called SyGlass (IstoVisio, United States) was used to visualize the internal structures of all five hearts in 3D and SyGlass was also used for making videos (Video are not included due to exceeded number of videos permitted by the journal but can be obtained upon request from the corresponding author). For each heart, the videos were taken through the chambers utilizing the same sequences. They were taken from the right side first, entered from the SVC into the RA, and observed superiorly to show the SVC, CT, and pectinate muscles. Then the right ventricle (RV) was entered through the tricuspid valve and went toward the apex. Next, we tracked along the RV outflow tract, through the pulmonary valve into the pulmonary artery and left the heart. Then the left atrium was entered through one of the pulmonary veins. After observing the LA appendage, we went through the mitral valve into the left ventricle and went toward the apex. Then, finally went along the LV outflow tract, through the aortic valve into the aorta and left the heart.

A mix reality device, Microsoft HoloLens, was used to view one heart and to create one holographic (Supplementary Video S11).

#### 2.2.6 3D printing

The process begins by refining the STL files representing the myocardium, cardiac conduction system (CCS), and aorta to reduce their sizes to 97,833 KB, 4,899 KB, and 10,488 KB, respectively. These optimized files are then converted into G-code instructions using Stratasys GrabCAD software. Utilizing a Stratasys J850—Digital Anatomy 3D, the printing setup accommodates multiple materials to allow for heterogenous prints with mixtures of up to 5 materials which ensures the production of anatomically accurate and visually compelling models. Material selection is crucial for achieving the desired characteristics of each anatomical component. The myocardium is printed using VeroUltraClear resin for its exceptional translucence, allowing clear visualization of internal structures. The CCS is crafted with hard-Cyan Vero material to provide opacity within the transparent myocardium, enhancing clarity and anatomical fidelity. For the aorta, flexible Tissue Matrix material replicates the mechanical properties of authentic human tissue allowing for physical TAVR placements. Support structures are generated using SUP706 support material, necessary for maintaining the structural integrity of intricate anatomical features during printing.

Post-printing procedures require support material to be manually removed using water jetting techniques to ensure thorough cleansing while safeguarding delicate anatomical structures. Final prints undergo further refinement through sanding procedures to enhance surface texture and eliminate imperfections. To improve visual clarity and minimize refraction, Rust-Oleum Triple Thick Clear Glaze is applied to the finished models, resulting in visually clear anatomical representations.

#### 2.2.7 Computational simulation of 3D anatomy

The non-MI heart, 0059, was used for this part of the study as a proof of concept. Cellular electrophysiology was described using the minimal model presented previously (15). In this model, the total ionic current is given by *I*_*ion*_ = *I*_*Na*_ + *I*_*to*_ + *I*_*CaL*_ + *I*_*Kr*_ + *I*_*Kur*_ + *I*_*K1.*_ There is no concentration homeostasis and the current formulations were selected for their simplicity; this helps to ensure efficient simulation and, in this context, that the resultant activation sequence and dynamics are a consequence of the structure only. Please see the supplement of the original publication (15) for full equations, which are beyond the scope of brevity in this present study. Parameters were set for each region to capture regional differences in the important features for activation and repolarization: the *APD*_90_ and *dVm/dt*_*max*_. See Supplementary Table S2 for full parameter differences between each region. Note that pacemaker regions do not exhibit spontaneous pacemaking in this simple model and, rather, were just set to exhibit a slower conduction velocity.

Tissue simulations were performed by down-sampling the segmented images by a factor of four, from 73 μm to 292 μm, before reconstruction into a 3D regular structured grid with dimensions 419 × 353 × 263 (NX × NY × NZ), resulting in 38,899,541 nodes of which 6,029,690 correspond to excitable cardiac tissue. Dynamics in the 3D tissue is described by the isotropic monodomain equation:


$$\frac{\partial V_{m}}{\partial t}=D\,\nabla^{2}V_{m}-\frac{I_{i\,o\,n}}{C_{m}}$$

Where *V*_*m*_ is the membrane potential, *D* is the diffusion coefficient (under a scalar approximation), *I*_*ion*_ is the total ionic current at each node and ∇ is the spatial Laplacian operator in 3D. The monodomain equation was discretized using a finite-differences method, centered differences approach using an integration space step corresponding to the spatial resolution of the down-sampled image data (Δx = 292 μm) and temporal step, Δ*t* = 0.01 ms. To impose the correct activation sequence, mediated by the CCS, regions at the superior and inferior end of the SN were segmented and set to be electrically coupled to the atria, whereas the remainder of the SN was coupled only to other SN cells. The AVN was set to be coupled only to the atria and the His-Purkinje system (i.e., it was insulated from the ventricles). Regions of the His-Purkinje system were crudely segmented as insertion points where they are coupled to the ventricles; elsewhere, the system is only electrically coupled to adjacent AVN or His-Purkinje system.

For normal conduction, a stimulus was applied to a region in the center of the SN. A stimulus was applied to a region of the His-Purkinje system for arrhythmic excitation. The diffusion coefficient describing isotropic diffusion was set to 0.3 mm^2^/ms, giving a conduction velocity in the working myocardium of 0.713 m/s. In the SN and AVN, the diffusion coefficient was set to 0.1 mm^2^/ms and the maximum upstroke velocity of the action potential model was reduced to reproduce qualitatively slower conduction in these regions. Conversely, the diffusion coefficient was increased to 0.9 mm^2^/ms in the His-Purkinje system and the crista terminalis/pectinate muscle/Bachmann’s bundle regions of the atria to increase conduction velocity along these conduction pathways. However, these parameters were only loosely matched, as the activation sequence, rather than activation times, was prioritized in this simple, illustrative model. Simulations were not fully optimized in this context, but were still feasible despite the very large dataset, with the model taking just over three hours for one second of simulation time on a 32-core system. This can certainly be improved in future.

## 3 Results

The tomographic slice-by-slice views of all five hearts from the xy, yz, and xz planes can be found in Supplementary Videos S1–S5. Supplementary Videos S6–S10 show the segmentation of all structures in each heart. The videos of the internal structures are not included due to exceeded number of videos permitted by the journal but can be obtained upon request from the corresponding author).

**FIGURE 1:**
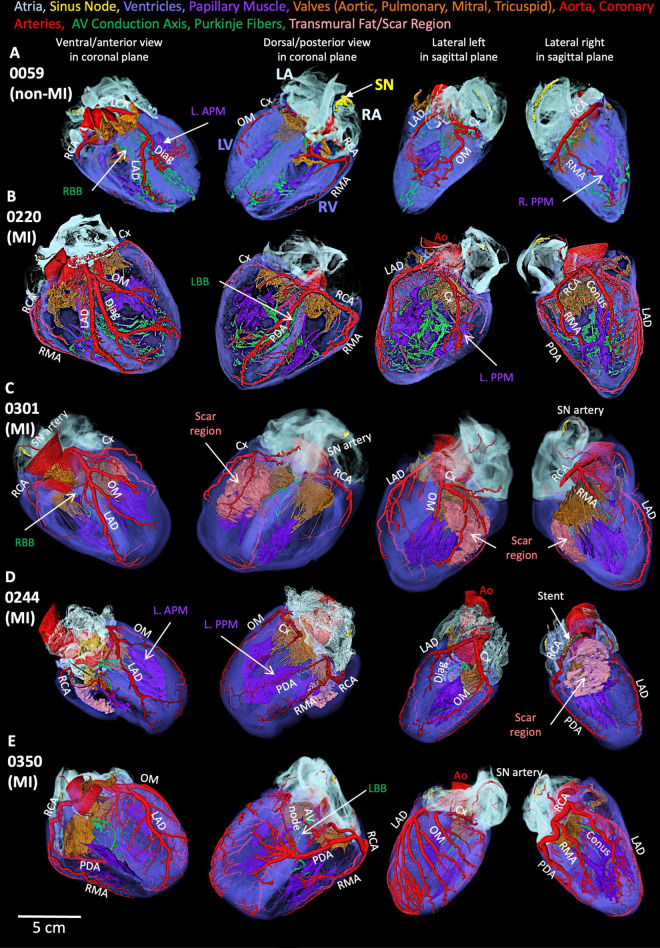
Segmented structures of five human hearts. **(A)** Non-MI heart 0059. **(B)** MI heart 0220 (MI). **(C)** MI heart 0301. **(D)** MI heart 0244 (MI). **(E)** MI heart 0350. Ao, aorta; Conus, Conus artery; Cx, circumflex artery; Diag, diagonal artery; LAD, left anterior descending artery; L. APM, left anterior papillary muscle; LCA, left coronary artery; LA, left atrium; L. APM, left anterior papillary muscle; LBB, left bundle branch; OM, obtuse marginal artery; L. PPM, left posterior papillary muscle; LV, left ventricle; PDA, posterior descending artery; RA, right atrium; RBB, right bundle branch; RCA, right coronary artery; RMA, right marginal artery; R. PPM, right posterior papillary muscle; RV, right ventricle; SN, sinus node.

We present the segmented structures in one non-MI heart and four MI hearts using contrast-enhanced non-destructive micro-CT technique (Figure 1), whereas Supplementary Figures S1, S2 show the segmentation of coronary arteries and CCS. The color codes of segmented structures are provided in Table 1 and within the figures and are used throughout this manuscript.

### 3.1 Cardiac conduction system in non-MI heart

**FIGURE 2:**
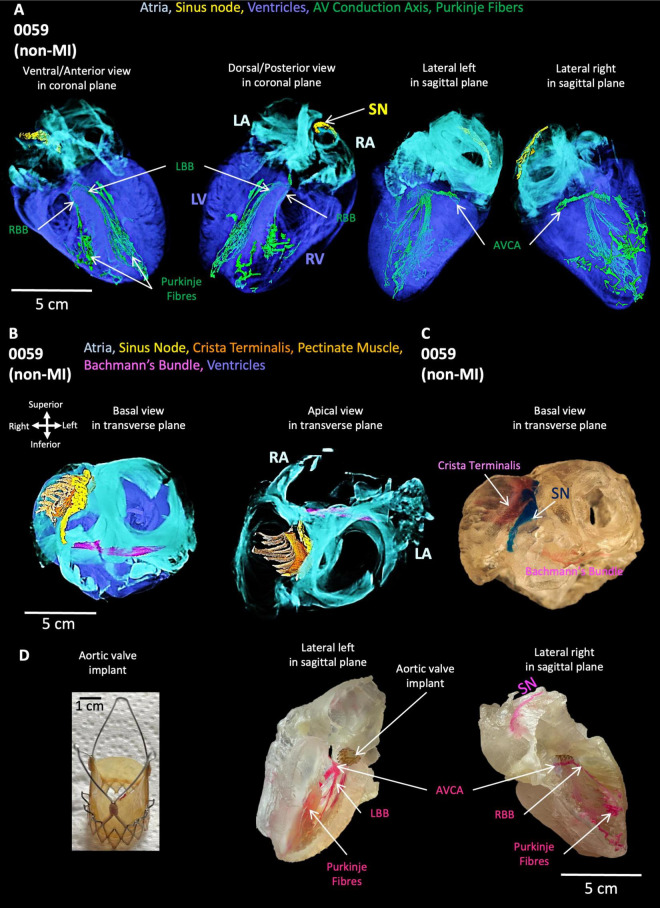
Segmentation of cardiac conduction system, Bachmann’s bundle, crista terminalis, pectinate muscle, atria and ventricles in the non-MI heart. **(A)** The cardiac conduction system within atria and ventricles. **(B)** Bachmann’s bundle, crista terminalis, pectinate muscle, and SN with atria. **(C)** 3D printing of Bachmann’s bundle, crista terminalis, pectinate muscle and SN with atria. **(D)** TAVI (left) and 3D printing of AVCA: atrioventricular conduction axis and Purkinje fibers. LA: left atrium; LBB, left bundle branch; LV, left ventricle; RA, right atrium; RBB, right bundle branch; RV, right ventricle; SN, sinus node; TAVI, transcatheter aortic valve implantation.

In the non-MI heart (0059), the SN is in the RA close to the SVC and along the CT toward the IVC (Figure 2A). In the same heart, the atrioventricular conduction axis and Purkinje fibers were also segmented and shown in Figure 2A. The AV conduction axis lies on top of the ventricular septum and travels from the posterior and superior to the anterior and inferior, then branches into left bundle and right bundle (LBB, RBB). LBB and RBB run on the surface of LV and RV endocardium and brunch into free-running Purkinje networks.

The segmented Bachmann’s bundle is also shown and travels from the CT close to the head of the SN and posterior to the aorta toward the LA (Figure 2B). The pectinate muscles are presented from the CT and run perpendicular to the CT toward the RA appendage (Figure 2B). The 3D-printed CT, SN, and Bachmann’s bundle are shown in Figure 2C, whereas the aortic valve implant (Figure 2D) is inserted in the printed CCS within the atria and ventricles (Figure 2D).

### 3.2 High-resolution 3D simulations of the non-MI heart

**FIGURE 3:**
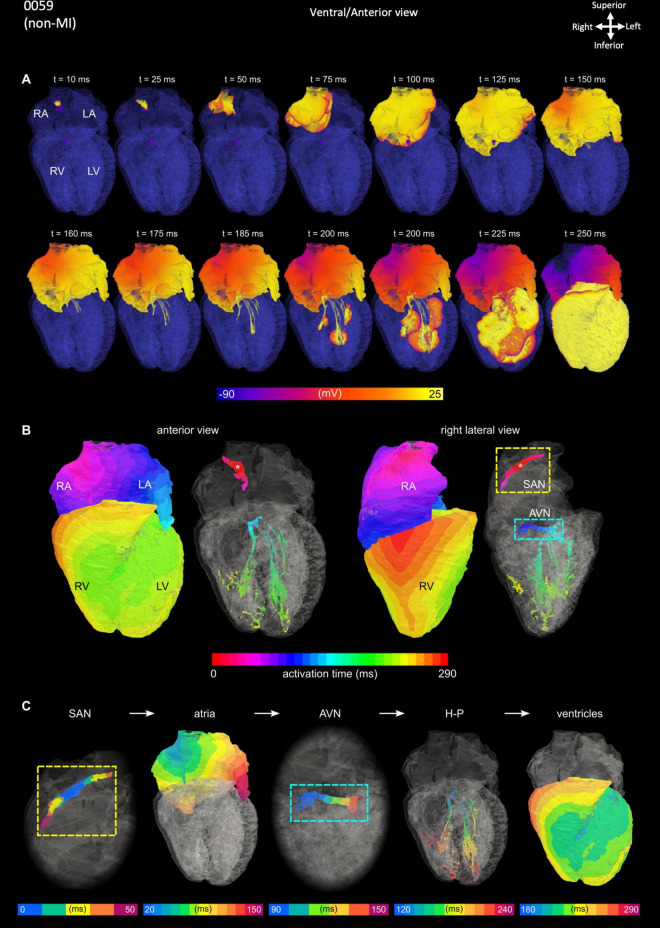
Simulation of normal activation sequence in the non-MI heart. **(A)** Temporal voltage snapshots during the normal activation sequence. *t* = 0 ms corresponds to the time of the applied stimulus. **(B)** Activation maps showing the surface myocardium and the isolated CCS, visualized from two views. The same color scale is used for all panels which covers the total range of activation time. **(C)** Localized activation maps for different regions of the SN, atria, AVN, His-Purkinje system, and ventricles. The colors are scaled to the total activation time of each region individually to give a higher level of temporal resolution for these localized activation sequences.

The simulated cardiac activation pattern qualitatively captures the conduction of the action potentials through heart 0059 (Figure 3; Supplementary Videos S12, S13). Excitation slowly propagates throughout the SN before exiting into the surrounding atria. The wavefront then propagates largely uniformly throughout the atria, except for where the CT, pectinate muscle or Bachmann’s bundle accelerate propagation. Excitation is then delayed as it transmits through the AVN to the His-Purkinje system, along which it rapidly propagates before exciting the ventricular myocardium quite uniformly. The far-right region of the RV shows the latest activation, which is primarily determined by the lack of a well-connected His-Purkinje system extending into this region in the reconstruction. It should be noted that the activation times have not been parameterized to match the clinical data. This qualitative pattern was considered normal, and the anatomical structure of the CCS controls this sequence. Further explanation of this figure and its panels is provided in the figure legend.

### 3.3 Simulation of abnormal activation using the non-MI heart

**FIGURE 4:**
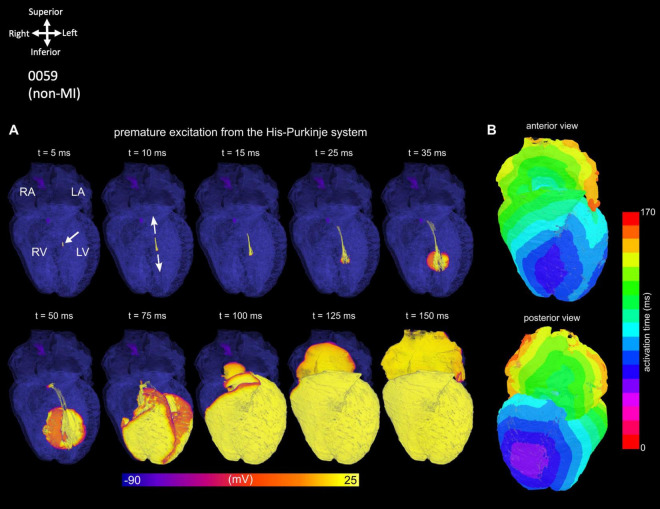
Simulation of an ectopic, focal excitation. **(A)** Temporal voltage snapshots. **(B)** Surface activation maps corresponding to the excitation pattern following a stimulus (white arrow) applied within the His-Purkinje system.

The simulation of arrhythmic pacing from the His-Purkinje system was performed on 0059 heart and shows a retrograde activation of the atria as the excitation wave transmits through the AVN (Figure 4) compared to the healthy situation (Figure 3; Supplementary Video 14). Further explanation of this figure and its panels is provided in the figure legend.

### 3.4 The identified CCS within the MI hearts

**FIGURE 5:**
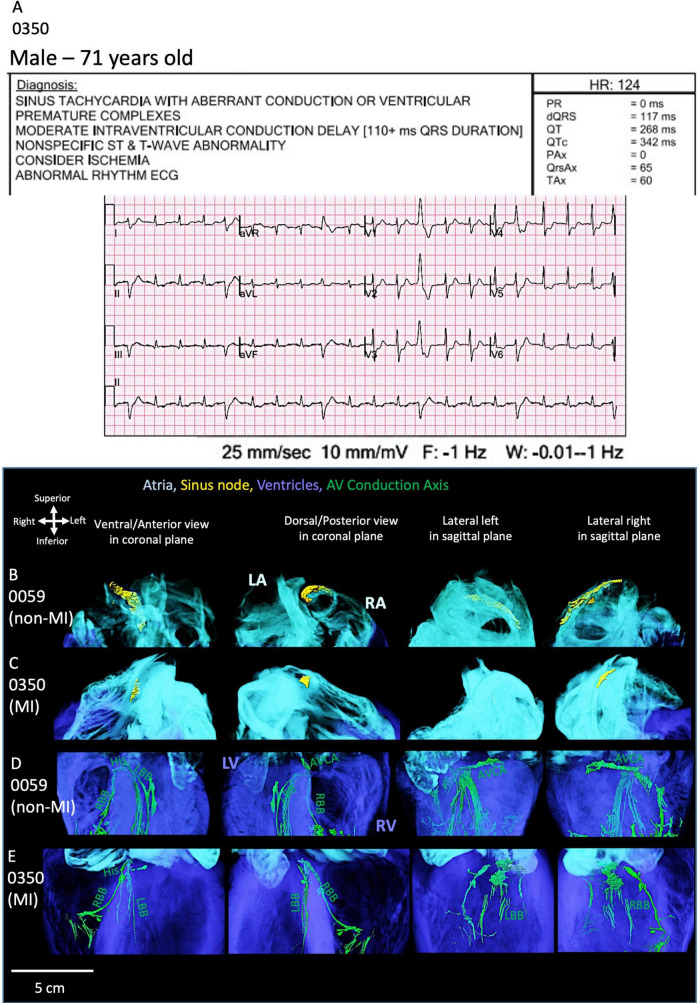
ECG and cardiac conduction system segmentation of one MI heart. **(A)** The 12-lead-ECG of the MI heart (0350) shows abnormal rhythm. The sinus node (SN) segmentation in the non-MI heart **(B)** is compared to the MI heart **(C)**. The atrioventricular conduction axis (AVCA) in the non-MI heart **(D)** is compared to the MI heart **(E)**. The MI heart shows atrophy of the SN and fragmented AVCA and thinning of bundle branches. AVCA, atrioventricular conduction axis; His, his bundle; LA, left atrium; LBB, left bundle branch; LV, left ventricle; RA, right atrium; RBB, right bundle branch; RV, right ventricle. AVCA includes the following structures: atrioventricular node, AVN, penetrating bundle, PB, His bundle, right and left bundle branches.

As shown in Figure 5A, one MI heart (0350) shows an ECG recording taken before the organ recovery with multiple rhythm abnormalities. The segmented SN in this MI heart is located more inferiorly in the intercaval region and is smaller compared to the non-MI heart (0059) (Figures 5B,C). In this MI heart, the atrioventricular conduction axis (ACVA) is also remodeled as compared to the non-MI heart (Figures 5D,E). The right and left bundle branches are disconnected and thinner compared to the non-MI heart.

These morphological changes observed in heart 0350 are consistent with what is observed in the ECG and the presence of a biventricular implantable cardioverter defibrillator (ICD). The segmentation of this device is shown in Supplementary Video S10.

The morphological alterations in the CCS seen in this specific MI heart (Figure 5; Supplementary Figures S2–S4) can also be observed in other MI hearts studied (especially 0301 and 0244 hearts; shown in Supplementary Figures S2–S4). All four MI hearts show significantly structurally smaller SN and AVCA (best illustrated in Supplementary Figures S3, S4).

Thinning of the left and right bundle branches (especially in 0301, 0244, and 0350 hearts) and more extensive Purkinje networks (heart 0220 best illustrated in Supplementary Figure S2) is evident.

The majority of MI hearts 0301, 0244, and 0350 were from hypertensive patients according to their medical histories (see Table 1). The measurements of the CCS are provided in Supplementary Figure S4 and Supplementary Table S2. Selected tomographic slices of the sinus node and atrioventricular conduction axis regions are shown in Supplementary Figure S5. Poor attenuation differences were obtained due to the high content of fat in these MI hearts, discussed further in section 5. The segmentation of the SN and AVCA structures was most likely not optimal.

### 3.5 Relationship of AV conduction axis, bundle branches and Purkinje networks with valves and papillary muscles

**FIGURE 6:**
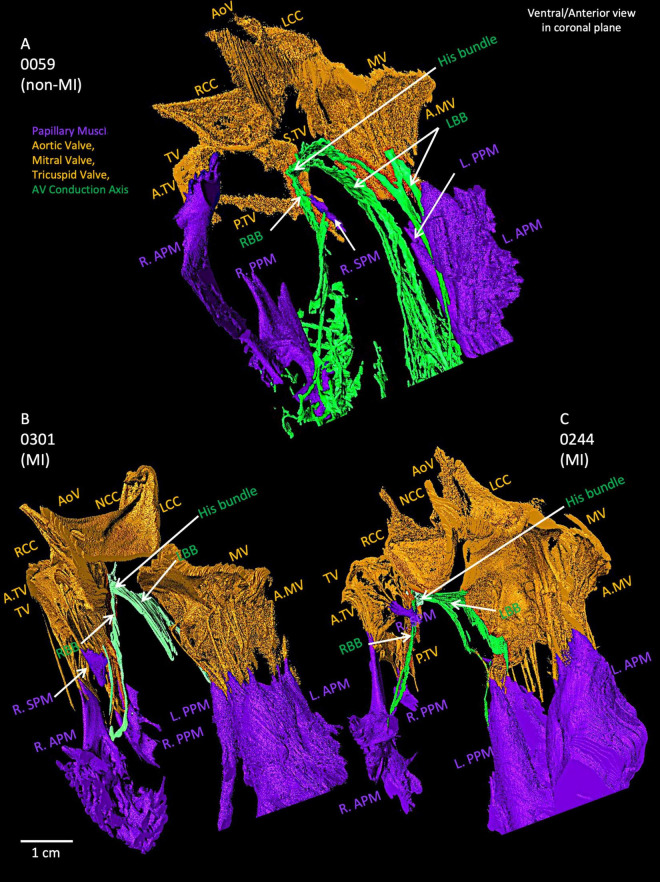
Closer view of segmented valves, papillary muscles and atrioventricular conduction axis in the non-MI heart **(A)** and two MI hearts **(B,C)**. The relationship of His bundle and its right and left bundle branches to the valves and papillary muscles is shown. AoV, aortic valve; A.MV, anterior cusp of the mitral valve; A.TV, anterior cusp of the tricuspid valve; L.APM, left anterior papillary muscle; LBB, left bundle branch LCC, left coronary cusp of the aortic valve; L.PPM, left posterior papillary muscle; MV, mitral valve; NCC, non-coronary cusp of the aortic valve; P.MV, posterior cusp of the mitral valve; P.TV, posterior cusp of the tricuspid valve; R.APM, right anterior papillary muscle; RBB, right bundle branch; RCC, right coronary cusp of the aortic valve; R.PPM, right posterior papillary muscle; R. SPM, right septal papillary muscle; S.TV, septal cusp of the tricuspid valve; TV, tricuspid valve.

As shown in Figure 6A, the AVCA in the non-MI heart is located close to all three valves (the AoV, MV and TV) with distances of less than 2 cm. The His bundle is in the inferior of the right coronary cusp (RCC) of the AoV.

Supplementary Figures S6A–D illustrates the penetrating bundle of the AVCA runs along the inferior extent of the membranous septum as it projects anteriorly. The proximal left bundle branch of the AVCA appears as a continuation of the His bundle, emerging as a broad fascicular structure projecting anteriorly and inferiorly along the crest of the ventricular septum and the junction with the membranous septum. The branching bundle and proximal left bundle branch of the AVCA are near the aortic root structures. The non-coronary aortic sinus is the most anteriorly located and is found to be the point with the least separation between tissues, with less than ∼6 mm from the most anterior and distal extension of the branching bundle to the plane of the aortic valvar attachments. The right coronary sinus overlies, lying ∼8 mm the branching bundle and the most proximal portion of the left bundle branch as it courses inferior to the membranous septum.

Figures 6B,C depicts the relationships of AVCA, valves and papillary muscles within two MI hearts. Compared to the non-MI heart in Figure 6A, the valves are thicker, and the papillary muscles are larger, (especially the tricuspid valve and left papillary muscles). The right posterior papillary muscle in the MI hearts is smaller than in the non-MI heart. In 0059, the LBB and RBB tend to project toward the posterior papillary muscles. In contrast, in the two MI hearts, the RBB tend to project toward the right anterior papillary muscle more. The bundle branches appear thinner compared to the non-MI heart and are reduced in volume (Supplementary Table S1).

**FIGURE 7:**
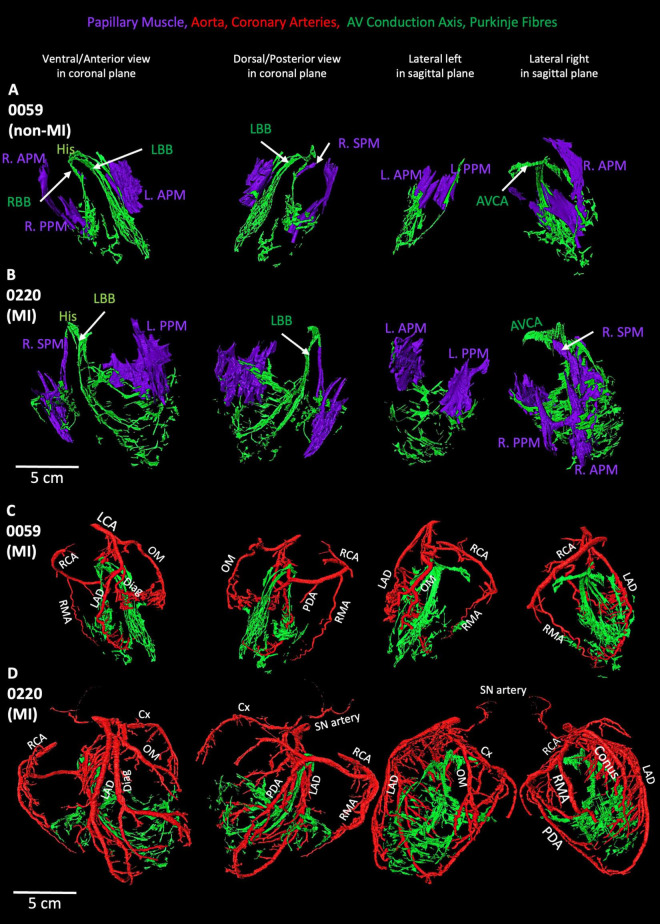
Segmented coronary arteries and papillary muscles, atrioventricular conduction axis, and Purkinje fibres in one non-MI heart and one-MI heart. **(A,B)** The relationship of papillary muscles to the AVCA and Purkinje fibres in one MI and one non-MI heart. **(C,D)** The relationship of coronary arteries to the AVCA and Purkinje fibres in one MI and one non-MI heart. AVCA, atrioventricular conduction axis; Conus, Conus artery; Cx, circumflex artery; Diag, diagonal artery; LAD, left anterior descending artery; L.APM, left anterior papillary muscle; LCA, left coronary artery; L.APM, left anterior papillary muscle; LBB, left bundle branch; OM, Obtuse marginal artery; L.PPM, left posterior papillary muscle; PDA, posterior descending artery; R.APM, right anterior papillary muscle; RBB, right bundle branch; RCA, right coronary artery; RMA, right marginal artery; R.PPM, right posterior papillary muscle; R.SPM, right septal papillary muscle.

In the MI heart (0220), the segmented AVCA and Purkinje network are compared with the non-MI heart (Figures 7A,B). In this MI heart, the Purkinje fibers network is extensive and stretched. Its Purkinje network volume is doubled than the Purkinje network volume (see Supplementary Table S1) in the non-MI heart, and both bundle branches are thinner and more stretched compared to non-MI hearts. Also, the Purkinje network in 0220 extensively overlays the papillary muscles (Figure 7B). This heart also shows that the valves are larger in volume yet (discussed below), and the papillary muscles are stretched and thinner (Supplementary Table S1; Figures 7B; Supplementary Figure S2B).

All segmented valves show complex anatomical changes illustrated in Supplementary Figures S7, S8).

### 3.6 Coronary arteries and their relationship with CCS, scar region and stent

As shown in Figures 1, 7 and Supplementary Figures S1, S9, S10, the morphology of the coronary arteries changed in the MI hearts compared to the non-MI heart. More coronary artery branches and collateral arteries can be observed in MI heart 0220 (best illustrated in Figure 7D). It is confirmed in the volume measurements, and the other three MI hearts all show increased coronary artery volume compared to the non-MI heart (Supplementary Table S1; Figure 1; Supplementary Figures S1, S9, S10).

In Figure 7D, extensive branches of coronary arteries match the vast Purkinje network in the MI heart 0220. Presumably to provide blood supply to the increased Purkinje network.

In two MI hearts (0301 and 0244) we were able to identify scar regions which were segmented together transmural and epicardial fat (Supplementary Figure S9). By comparison to the healthy heart (0059) with epicardial fat volume of 55.71 cm^3^, two MI hearts show increased epicardial fat volume (169.67 and 109.07 cm^3^ (Supplementary Figures S9C,D).

In another MI heart, 0244, the artificial stent can be segmented (Supplementary Figure S10). It aligns with the clinical coronary angiography data shown in Supplementary Figure S10A. In this heart, the increased collateral arteries can also be identified presumably to provide increased blood supply to the increased epicardial fat layer (refer to Supplementary Figure S9 and Supplementary Table S1 for detailed epicardial fat segmentation and measurements).

## 4 Discussion

In our initial research on this topic, we published high-resolution anatomical details of the CCS of the non-MI *ex-vivo* human heart (0059) using the contrast-enhanced micro-CT technique (11). Subsequently, we compared human CCS anatomy in an *ex-vivo* heart from an elderly organ donor and one from an *ex-vivo* heart from an obese patient using the same technique (12). In the present study, not only did we further analyze the anatomy for this previously described healthy *ex-vivo* specimen (0059) but added additional diseased *ex-vivo* hearts. Along with detailed reconstructions of the various CCS anatomy, we also included major valves, coronary arteries, papillary muscles, pectinate muscles, CT, and Bachmann’s bundle. All structures were segmented from four *ex-vivo* MI hearts to further determine the precise anatomical positions of the CCS components and their inter-relationships among these important cardiac structures. Interesting differences in the CCS-generated anatomical models and the major cardiac structures were observed and described in this study and then used to develop novel educational tools (see all videos).

The proximity of the AVCA and the aortic root structures has been observed previously using CT and classical histology (16). The use of 3D imaging allows the interrelationship to be more clearly observed in this clinically important and variable anatomical region. It has been reported to be variability in the membranous septal morphology, affecting the observed course of the AVCA (16). In the recently published article, other types of micro-CT techniques have been used to elegantly visualize 3D cardiac anatomy in *ex-vivo* hearts, for example, superior hierarchical phase-contrast tomography (HiP-CT) with a voxel size of around 20 μm, and the resolution as low as 2.3–6.4 μm (17).

In our study, MI heart 0220 shows an increase in the LV chamber diameter of more than 5 cm, along with the thinner ventricular free walls (and with a low ejection fraction of less than 40%)—these parameters commonly correspond to the diagnosis of dilated cardiomyopathy (2). As Figures 1, 7 and Supplementary Figures S1, S9, S10 show, MI hearts have extensive and increased coronary artery volumes compared to the non-MI heart. The increased number of coronary arteries and vast forming of collateral arteries indicate a required increased blood supply in these MI hearts. Such an increased blood supply is considered necessary for the increased energy requirements from altered morphologies, as also represented by an increased Purkinje network, working myocardium, valves and papillary muscles volume and more epicardial fat in MI hearts.

In our study, we found the proximal branch of the left coronary artery (LCA) prior to branching for the left anterior descending (LAD) to be the main source of blood supply to the anterior ventricular septum in the region of the proximal LBB and RBB (shown in Supplementary Figure S6E). This vessel undergoes extensive branching within the muscular septum, and we were unable to trace the vessels to allow us to ascertain whether each fascicle has an independent blood supply. The circumflex and diagonal branches supply the myocardium and, presumably, the underlying conduction fibers, although we were unable to trace branches to this extent (Supplementary Figure S6E). This is consistent with what has been observed histologically (18).

It has been reported that an occlusion of the left anterior descending coronary artery can lead to a left anterior fascicular block (19) and an acute proximal occlusion of the right coronary artery, together with proximal obstruction of the left anterior descending coronary artery, can lead to a left septal fascicular block (20).

As our present research has shown, the SN in MI hearts tends to be located more inferiorly along the CT, which aligns with previously described as the so-called the “wandering pacemaker.” This can lead to a benign atrial arrhythmia that is commonly seen in elderly patients. The main pacemaker site wanders from the SN to the RA and the atrioventricular junction with a changing focus, causing the different P wave configurations on the ECGs (21, 22). It has been reported that regional myocardial ischemia or injury may affect the CCS blocks at various locations. For example, the left bundle branch block is associated with multi-vessel disease, whereas the right bundle branch block indicates proximal occlusion of the left anterior descending coronary artery (23). In acute or chronic MI, idiopathic left VT can arise from the left Purkinje fibers, and this type of VT can be terminated by catheter ablation without affecting left ventricular conduction (24).

The high-resolution segmentations of the MI scar regions and/or the implanted clinical devices *in ex-vivo* hearts (see Supplementary Figure S9 and Supplementary Videos S8–S10) show the investigative potential applications. For example, using micro-CT on post-mortem *ex-vivo* hearts to study the impacts of post-lead implantations on surrounding cardiac structures, could lead to improved treatment strategies (10). Currently, in cardiac electrophysiology clinics, a major difficulty for arrhythmia management continues to be lead management, with lead dislodgement or lead displacement as the most common complication. The displacement rates remain around 1–5% depending on the pacemaker type and many occur within the first 6 weeks after the procedure, leading to the requirement of reintervention. The atrial leads are affected more frequently than the ventricular lead (25, 26). The repositioning of leads or the implantation of a new lead increases the chance of tricuspid valve structural and/or functional damage, such as valvular stenosis and valve regurgitation (27, 28). The 3D model of the segmented device, CCS, valves, papillary muscles, atria, and ventricles from the *ex-vivo* heart provided here in our novel research (e.g., Supplementary Video S10) may help to improve surgical procedures thus potentially reducing the requirements of reintervention and improving clinical outcomes.

The detailed computational segmentations performed in our study also identified changes in 3D valve anatomy in the MI hearts (Supplementary Videos S6–S10). Aortic, mitral, and/or tricuspid valve repairs and replacements carry major risks of post-procedure high-grade atrioventricular blocks requiring pacemaker implantation (29). Improving understanding of these complex cardiac anatomy as presented in our *ex-vivo* post-mortem MI hearts alongside functional changes could be used in the development of improvements to valve replacements, repairs, or novel mechanical valve devices. The 3D anatomy models of *ex-vivo* hearts generated in this study of these valve regions provide novel high-resolution visualization tools for clinicians and medical device developers. For example, the close anatomical relationships between the valves and LBB and RBB in the human heart are shown in Figure 6.

There is great clinical interest in the anatomy of the LBB and RBB and His bundle for clinical pacing; especially with the development of new tools and strategies for lead placements in these anatomical locations (30). Traditionally, a patient’s ventricular pacing lead is positioned in the apical or septal ventricular myocardium, but this method is likely to produce ventricular dyssynchrony that may in turn lead to mitral and tricuspid valve regurgitation, atrial fibrillation, as well as systolic contractile dysfunction (31, 32). Noteworthy, the death or heart failure hospitalization rate at 5 years is around 50% in patients done ventricular pacing (33). His bundle pacing has shown to have improved clinical outcomes compared to ventricular pacing, including lower HF hospitalization rates, low event rates, and low death rates (33, 34). Nevertheless, His pacing requires the accurate implantation of the pacing lead increasing the complexity of such an intervention (35). An enhanced understanding of the His bundle and LBB and RBB anatomies in the healthy *ex-vivo* heart and the changes seen in disease *ex-vivo* can aid the needed anatomical research to further improve the His bundle pacing and other potential stimuli delivery systems.

Super high-resolution 3D computational models and 3D prints are increasingly being used in medical education and clinics to promote more accurate, precise, and accessible anatomical understandings. Several reported studies have described the uses of these techniques to create 3D models for patients with various cardiovascular diseases, mainly aortic diseases due to the complex anatomical features, such as transcatheter aortic valve replacement and endovascular stenting (8, 10, 36). These anatomical approaches show the feasibility of transferring the 3D reconstructed cardiovascular structures into wider applications such as virtual reality, mixed reality, and personalized medicine.

We demonstrated the feasibility of using the complex anatomical segmentation generated in this study in the computational modeling of cardiac activation. Computational simulation is a valuable tool for identifying potential mechanisms underlying normal cardiac function and disease-related dysfunction. The construction of a whole human heart model that accounts for all these features is a time-consuming endeavor that is beyond the scope of this study but is currently being worked on by project collaborators. Such a model, including all these factors, would be suitable for a hugely broad range of detailed simulation studies that can investigate electrophysiological and structural mechanisms of multiple cardiovascular conditions including, but not limited to, dysfunctions caused by CCS. The future incorporation of anatomical models of diseases, such as those presented in this study, into a detailed model of the whole heart, would enable highly rigorous analyses of the relationships between structural and electrophysiological remodeling in the mechanisms of various cardiovascular dysfunction.

## 5 Limitations

In this study, only the classic components of the CCS were identified; the peripheral structures, such as the paranodal area and AV ring tissue, were not segmented due to the close attenuation differences of these structures with the surrounding working myocardium in MI hearts. In MI hearts extensive amount of epicardial fat produced poor attenuation differences due to the contrast medium being trapped in this fatty tissue and not being able to penetrate deeper. As a result, scar regions and complete AVCA could not be determined in some MI hearts. It has been shown previously that epicardial fat accumulation causes atrial conduction abnormality (37).

We are aware that the 3D models are only from the limited number of post-mortem hearts, which cannot be used in generalized clinical determinations, especially for MI hearts. The hearts are in various conditions and heterogeneity. Acquired structural defects can affect valves, ventricular muscle thickness, cavities, and major arteries. We have limited information on the medical history of the patients for whom the hearts were used in this study (Table 1). It is possible that they have acquired a structural heart deformation due to high blood pressure, atherosclerosis, heart attack, aging, obesity, and any unknown other diseases.

Our developed simulations were isotropic and did not account for myocyte fiber orientation. It should be noted that fiber structures that underlie conduction antitropy will have a substantial impact on conduction patterns, for example, playing roles in the transmission of excitation from the right to the left atrium. Also, only a simple cardiac cell model was implemented, and electrophysiology in the SN and AVN regions was not autorhythmic. Segmentation of the SN exit pathways and coupling locations between the ventricles and His-Purkinje system were only crudely implemented in the present study. Future refinement of these segmentations from detailed analyses of the imaging data, influenced by functional mapping data, would substantially improve the reconstructed activation sequences. Action potential depolarization gradients across the atria, transmural ventricular walls, and within inter-ventricular heterogeneities were not all included, and thus, the repolarization sequences (and, hence, the dispersion of repolarizations) were not matched in our present study. Furthermore, details of physiological fibrosis distributions were also not included.

## 5 Conclusion

Novel micro-CT anatomical techniques can be used to visualize both healthy and diseased human hearts to study complex 3D cardiac anatomy. Importantly, the human heart after an MI can clinically undergo complex anatomical changes of the major structures including the working myocardium, vasculature, valves, and the CCS. The high-resolution 3D anatomical models presented here offer exciting new tools for medical education, clinical applications, computational simulations, and/or insights for personalized/precision medicine. The utilization of these novel and innovative educational images and tools (see Supplementary Videos S11–S14) can potentially lead to improvements in disease diagnoses, new treatments, and/or medical device innovations.

## Figures and Tables

**Table:** 

AC	anterior cusp
AoV	aortic valve
AS	atrial septum
AVCA	atrioventricular conduction axis
CN	compact node
Conus	Conus artery
Cx	circumflex artery
Diag	diagonal artery
His	His bundle
INE	inferior nodal extension
L. APM	left anterior papillary muscle
L. PPM	left posterior papillary muscle
LAD	left anterior descending artery
LBB	left bundle branch
LC	left cusp
LCA	left coronary artery
LCC	left coronary cusp
MV	mitral valve
NCC OM	non-coronary cusp Obtuse mariginal artery
PB	penetrating bundle
PC	posterior cusp
PDA	posterior descending artery
PulmV	pulmonary valve
R. APM	right anterior papillary muscle
R. PPM	right posterior papillary muscle
R. SPM	right septal papillary muscle
RBB	right bundle branch
RC	right cusp
RCA	right coronary artery
RCC	right coronary cusp
RMA	right marginal artery
SC	septal cusp
SN	sinus node
TV	tricuspid valve
VS	ventricular septum

## Data Availability

The raw data supporting the conclusions of this article will be made available by the authors, without undue reservation.

## Footnotes

1
http://www.vhlab.umn.edu/atlas/histories/histories.shtml
