# Supplementary material for: High-resolution 3D visualization of human hearts with emphases on the cardiac conduction system components—a new platform for medical education, mix/virtual reality, computational simulation
Source: Front Med (Lausanne). 2025 Feb 18;12:1507005. doi: 10.3389/fmed.2025.1507005 (PMC11878103; doi:10.3389/fmed.2025.1507005)

High-resolution 3D visualisation of human hearts with emphases on the cardiac conduction system components – a new platform for medical education, mix/virtual reality, computational simulation

**Weixuan Chen^1^**, Marcin Kuniewicz^1,2^, Abimbola J Aminu^1^, Irem Karaesmen^1^, Neal Duong^3^, Klaudia Proniewska^2^, Peter van Dam^2,4^, Tinen L Iles^5^, Mateusz K Hołda^1,2^, Jerzy Walocha^2^, **Paul A Iaizzo^3#^**, **Michael Colman^6^**^#^, **Halina Dobrzynski^1,2#^, Andrew J Atkinson^1#^**

**#Join last**

^1^ Division of Cardiovascular Sciences, School of Medical Sciences, University of Manchester, Manchester, UK

^2^ Jagiellonian University Medical College, Krakow, Poland

^3^ The Visible Heart® Laboratories and the Institute for Engineering in Medicine, University of Minnesota, USA

^4^ University Medical Centre Utrecht, Netherlands

^5^ University of Minnesota, Department of Surgery, USA

^6^ The University of Leeds, Leeds, UK.

**Correspondence to:**Corresponding Author
halina.dobrzynski@manchester.ac.uk

Keywords: Cardiac Conduction System, micro-CT, computational simulation, coronary arteries, 3D printing and reconstruction, myocardial infarction, mix/virtual reality, visualisation

**Supplement materials**

**Table S1. Summary of measurements of different cardiovascular structures**

| Heart number | 0059 | 0220 | 0301 | 0244 | 0350 |
| --- | --- | --- | --- | --- | --- |
| RVFW thickness  (cm) | 0.57 | 0.36 | 0.50 | 0.63 | 0.65 |
| VS thickness  (cm) | 1.22 | 0.71 | 1.28 | 1.24 | 1.43 |
| LVFW thickness  (cm) | 1.34 | 0.65 | 1.15 | 1.28 | 2.06 |
| LV cavity width  (cm) | 2.54 | 6.41 | 2.41 | 1.97 | 2.69 |
| RV cavity width  (cm) | 3.06 | 2.45 | 2.67 | 1.44 | 3.41 |
| Atrial volume (excluding cavities)  (cm^3^) | 30.70 | 42.31 | 70.62 | 28.18 | 40.46 |
| Ventricular volume (excluding cavities)  (cm^3^) | 167.40 | 382.78 | 353.80 | 248.25 | 329.05 |
| Mitral valve volume  (cm^3^) | 1.32 | 2.01 | 2.09 | 0.87 | 0.87 |
| Tricuspid valve volume (cm^3^) | 0.41 | 1.12 | 1.14 | 0.28 | 0.74 |
| Aortic valve volume  (cm^3^) | 0.48 | 0.71 | 0.70 | 0.33 | 0.44 |
| Pulmonary valve volume  (cm^3^) | 0.24 | 0.52 | 0.24 | 0.12 | 0.21 |
| Coronary artery volume  (cm^3^) | 1.84 | 8.82 | 3.13 | 2.17 | 5.97 |
| Right bundle branch volume  (cm^3^) | 0.35 | 0.24 | 0.03 | 0.07 | 0.10 |
| Left bundle branch volume  (cm^3^) | 0.63 | 0.52 | 0.16 | 0.14 | 0.10 |
| Purkinje fibres volume  (cm^3^) | 0.67 | 1.49 | - | - | - |
| Voxel size  (µm) | 73.00 | 250.00 | 88.66 | 69.10 | 85.54 |

**Table S2: Summary of regional current and action potential parameters in the minimal model**

| Celltype | *g*_Na_ | *g*_to_ | *g*_CaL_ | *g*_Kur_ | *g*_Kr_ | *g*_K1_ | APD_90_ | dV/dt_max_ |
| --- | --- | --- | --- | --- | --- | --- | --- | --- |
| “Pacemaker” (SAN/AVN) | 5.67 | 0.1652 | 2.25 | 0.0053 | 0.15 | 0.15 (+20mV shift) | 185 ms | 87 mV/ms |
| Atria | 8 | 0.2891 | 0.56 | 0.0194 | 0.125 | 0.15 | 178 ms | 199 mV/ms |
| EPI | 8 | 0.081 | 2.25 | 0 | 0.15 | 0.15 | 282 ms | 203 mV/ms |
| M | 8 | 0.081 | 2.5 | 0 | 0.15 | 0.15 | 365 ms | 203 mV/ms |
| ENDO | 8 | 0.081 | 2.5 | 0 | 0.15 | 0.15 | 293 ms | 203 mV/ms |
| PK | 16 | 0.081 | 2.5 | 0 | 0.125 | 0.15 | 342 ms | 305 mV/ms |

**NOTE:** these are intended to be rough only, and reproduce the relationship of key factors between different regions, rather than being robustly parameterized.

**Figure S1. Main coronary arteries and their branches segmentation in the context of atria and ventricles.** Conus: Conus, artery Cx: circumflex artery, Diag: diagonal artery, LAD: left anterior descending artery, LA: left atrium, LCA: left coronary artery, LV: left ventricle, OM: obtuse marginal artery, PDA: posterior descending artery, RA: right atrium, RCA: right coronary artery, RMA: right marginal artery, RV: right ventricle, SN: sinus node.

**Figure S2. The sinus node, atrioventricular conduction axis segmentation in the context of atria and ventricles.** AVCA: atrioventricular conduction axis, LA: left atrium, LV: left ventricle, RA: right atrium, RV: right ventricle. AVCA includes the following structures: atrioventricular node, AVN, penetrating bundle, PB, His bundle, and right and left bundle branches.

**Figure S3. Segmented sinus node and atrioventricular conduction axis with segmented atria.** The Bachmann’s bundle is identified and labelled with the pink dashed lines in the apical view in the transverse plane. LA: left atrium, RA: right atrium.

**Figure S4. Segmented sinus node and atrioventricular conduction axis.** AVCA: atrioventricular conduction axis, AVN: compact node and penetrating bundle, His: His bundle, INE: inferior nodal extension, SN: sinus node.

**Figure S5. Selected tomographic slices of the sinus node and atrioventricular conduction axis regions.** The SN is outlined by the yellow dashed lines and yellow arrows. The compact node, penetrating bundle and His bundle are indicated by the green, red, and blue arrows, respectively. AS: atrial septum, CN: compact node, CT: crista terminalis, HIS: His bundle, PB: penetrating bundle, SN: sinus node, VS: ventricular septum.

**Figure S6. Proximity of the atrioventricular conduction axis with the membranous septum, aortic sinuses and coronary arteries. A.** Segmented AVCA, aorta and membranous septum from heart non-MI heart (0059) viewed along the plane of the right coronary sinus. **B.** Segmented AVCA, aorta and membranous septum from the non-MI heart (0059) viewed along the plane of the non-coronary sinus. **C.** Tomographic slice from plane of right coronary sinus used in A. **D.** Tomographic slice from plane of non-coronary sinus used in B. **E.** Arterial supply to the anterior/ventral portion of the interventricular septum (heart 0220). * denotes the septal branch proximal to LAD. Ao: aorta, CN: compact node, Cx: circumflex artery, INE: inferior nodal extension, LAD: left anterior descending artery, LBB: left bundle branch, LV: left ventricle, RBB: right bundle branch, RV: right ventricle.

**Figure S7. Segmented mitral and tricuspid valves and their relationship with papillary muscles.** AC: anterior cusp, L.APM: left anterior papillary muscle, L.PPM: left posterior papillary muscle, MV: mitral valve, PC: posterior cusp, R. APM: right anterior papillary muscle, R.PPM: right posterior papillary muscle, R.SPM: right septal papillary muscle, SC: septal cusp, TV: tricuspid valve.

**Figure S8. Aortic and pulmonary valve segmentation.** AC: anterior cusp, AoV: aortic valve, LC: left cusp, LCC: left coronary cusp, NCC: non-coronary cusp, PulmV: pulmonary valve, RC: right cusp, RCC: right coronary cusp.

**Figure S9. Epicardial fat and transmural fat/scar region segmentation from two MI hearts. A.B.** Segmentation of structures from two MI hearts to show the location of the transmural fat/scar region. **C.D.** The transverse view of epicardial fat and transmural fat/scar region. Cx: circumflex artery, Diag: diagonal artery, LAD: left anterior descending artery, LCA: left coronary artery, LA: left atrium, LV: left ventricle, OM: obtuse marginal artery, PDA: posterior descending artery, RA: right atrium, RCA: right coronary artery, RMA: right marginal artery, RV: right ventricle, SN: sinus node.

**Figure S10. Coronary arteries segmentation of one MI and one non-MI heart. A.** Schematic diagram of coronary angiography of MI heart 0244. **B.C.** Coronary arteries segmentation of one non-MI heart (B) and one MI heart (C) to show the stent. The blue arrows point to the segmented stent. Cx: circumflex artery, Diag: diagonal artery, LAD: left anterior descending artery, LCA: left coronary artery, OM, obtuse marginal artery, RCA: right coronary artery, RMA: right marginal artery.

Video S1. Tomographic slice view of micro-CT scanned Heart 0059.

Video S2. Tomographic slice view of micro-CT scanned Heart 0220.

Video S3. Tomographic slice view of micro-CT scanned Heart 0301.

Video S4. Tomographic slice view of micro-CT scanned Heart 0244.

Video S5. Tomographic slice view of micro-CT scanned Heart 0350.

Video S6. Segmented all structures of Heart 0059 related to Figure 1A.

Video S7. Segmented all structures of Heart 0220 related to Figure 1B.

Video S8. Segmented all structures of Heart 0301 related to Figure 1C.

Video S9. Segmented all structures of Heart 0244 related to Figure 1D.

Video S10. Segmented all structures of Heart 0350 related to Figure 1E.

Video S11. Holographic visualisation of Heart 0220 using Microsoft HoloLens.

Video S12. Heart 0059 shows a computational simulation of normal activation from multiple views: anterior view (left), right lateral view (middle) and a view looking down from the top (right), showing atrial activation (top) and with the atria removed to show ventricular activation (bottom).

Video S13. Segmented Heart 0059 within the thorax with computational simulation of normal activation.

Video S14. Heart 350; showing a computational simulation of abnormal/delayed ventricular conduction. The lack of the His-Purkinje system extending into the LV leads to a substantially increased ventricular activation time and abnormal activation sequence.


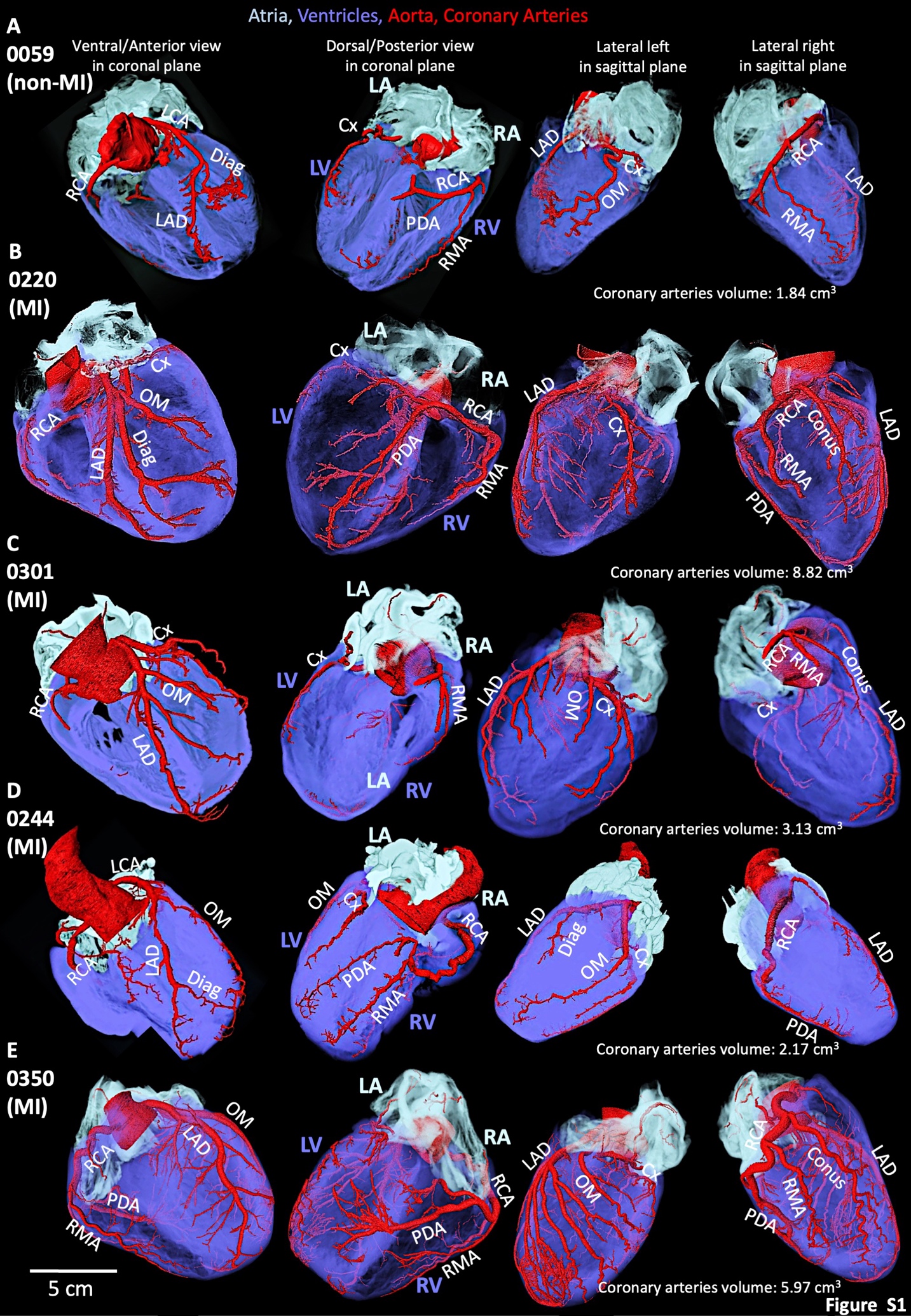

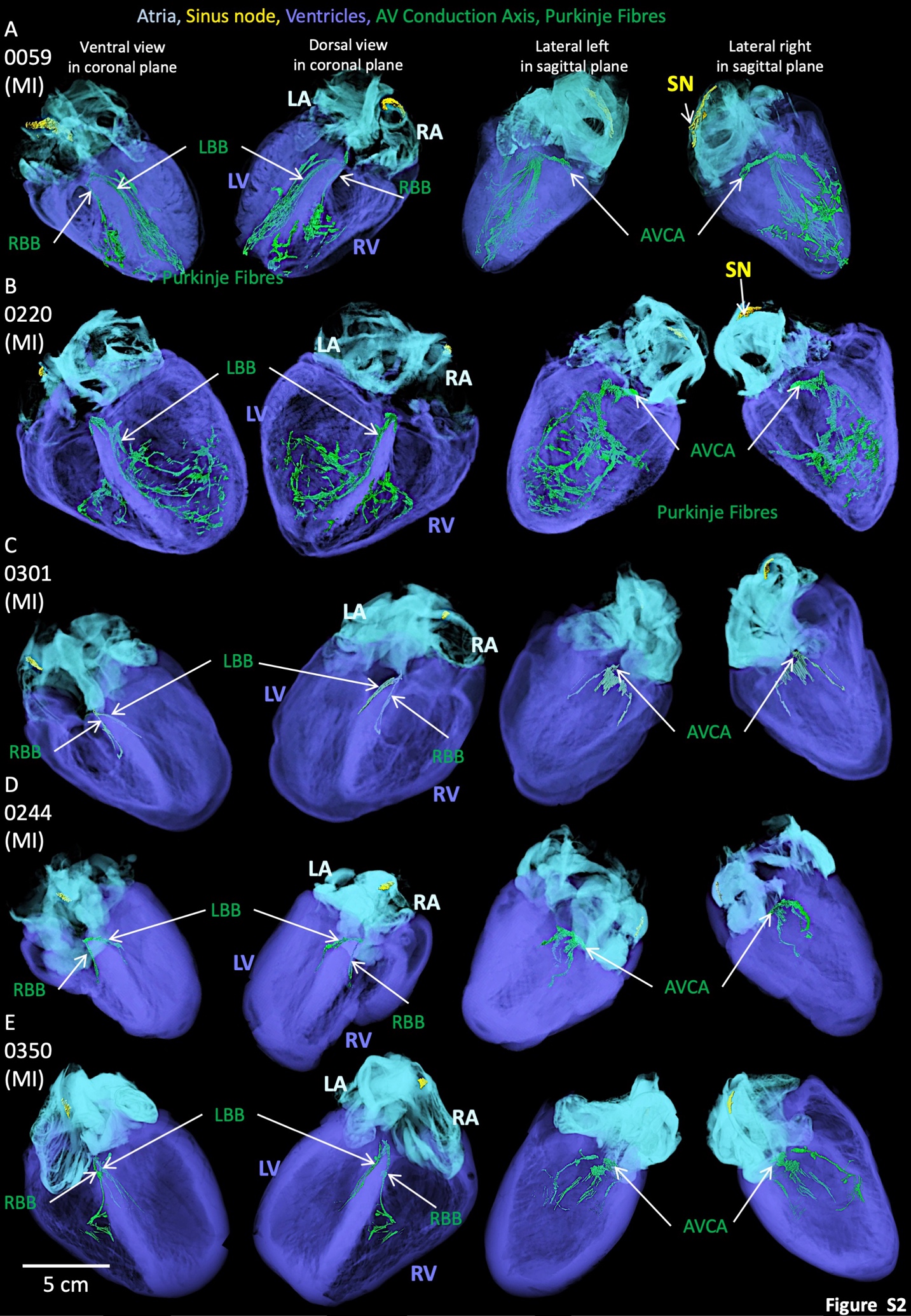

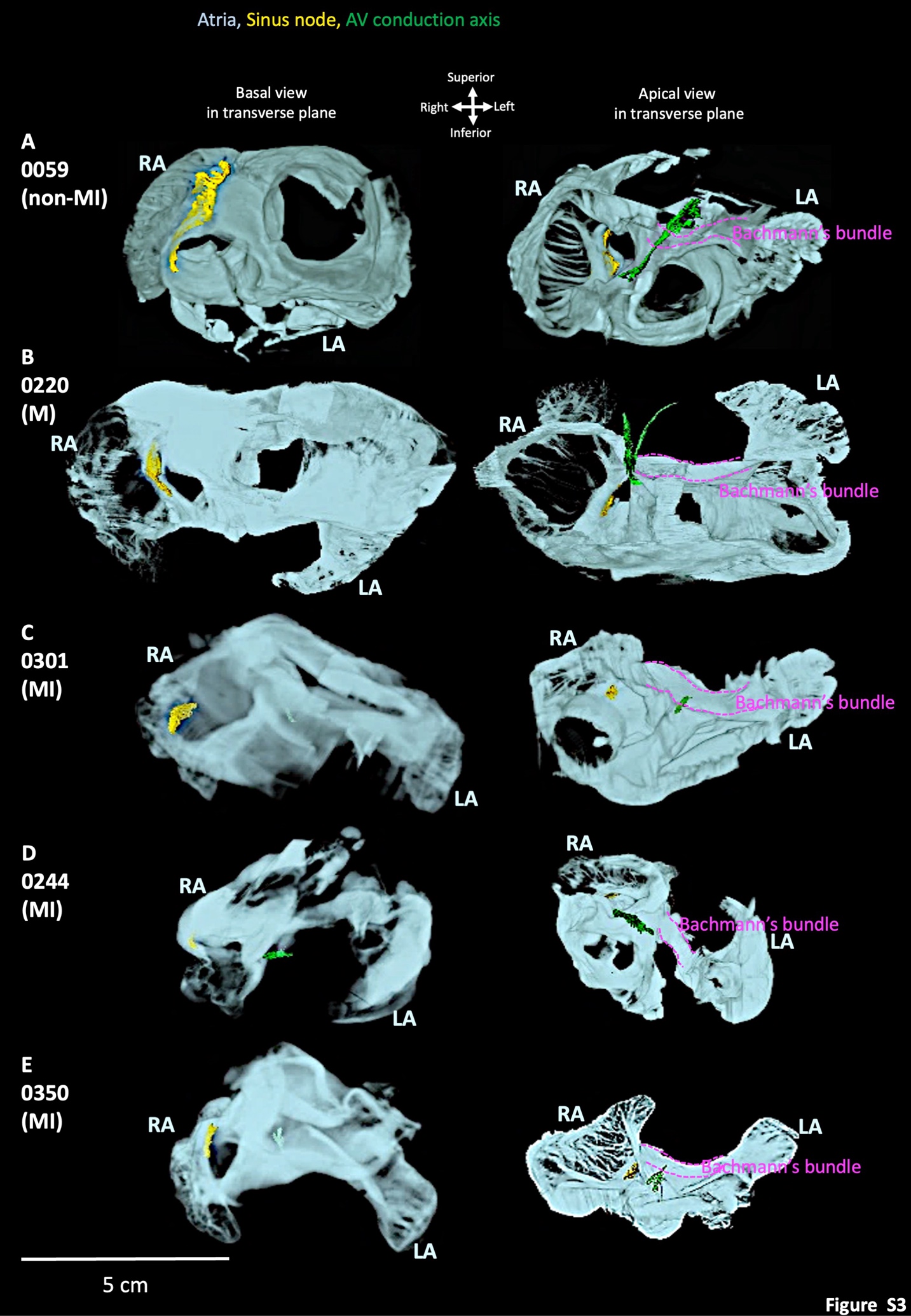

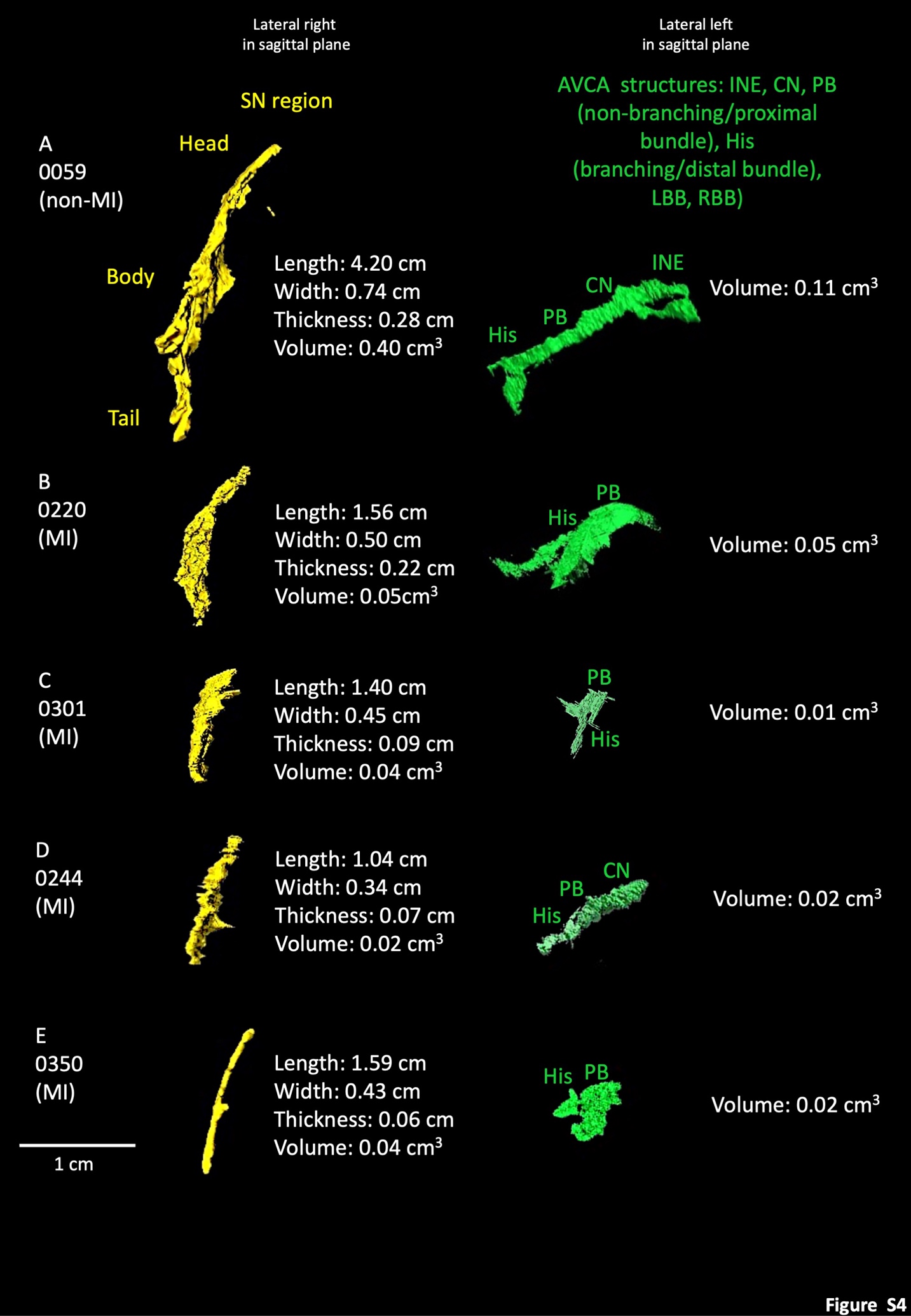

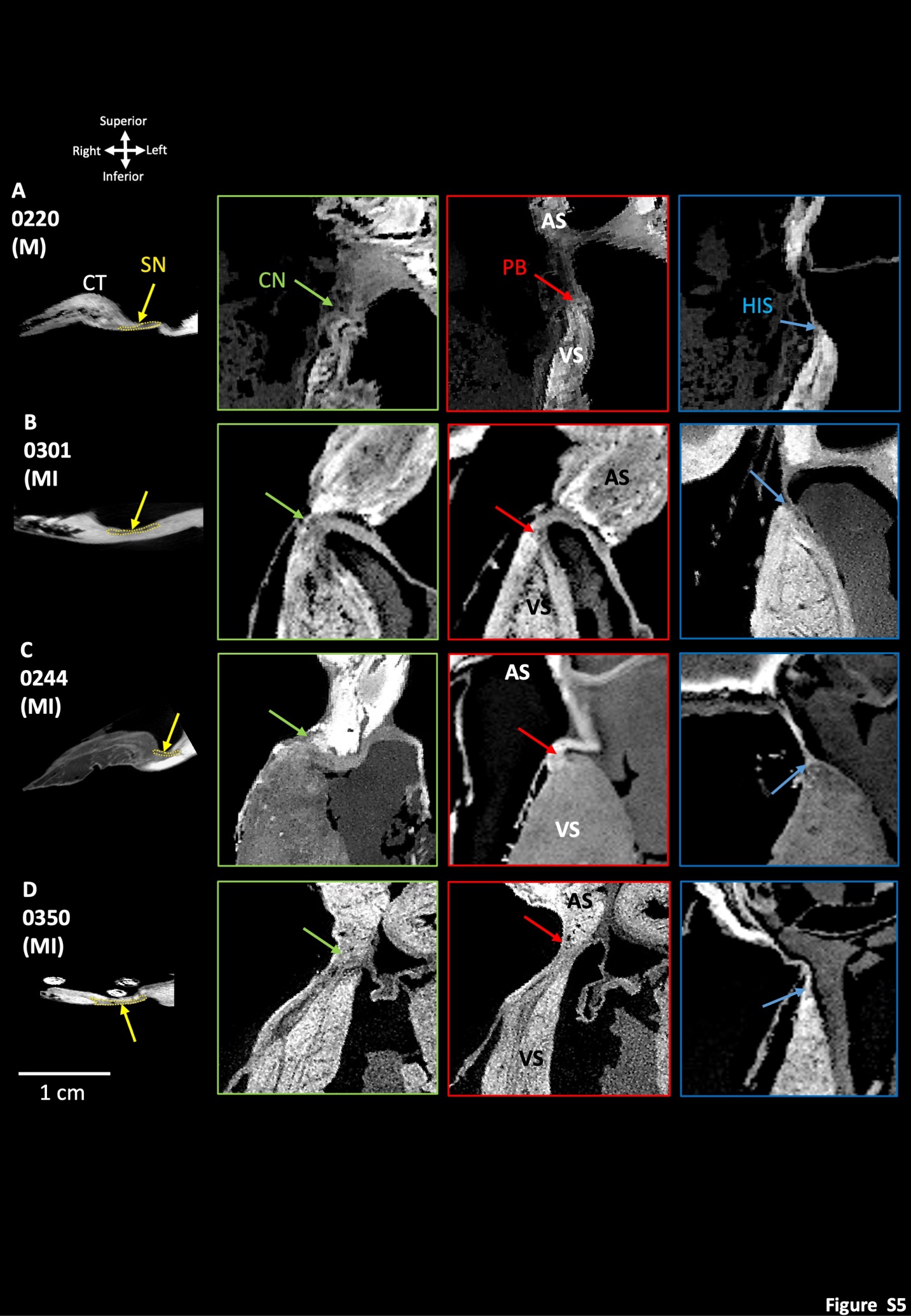

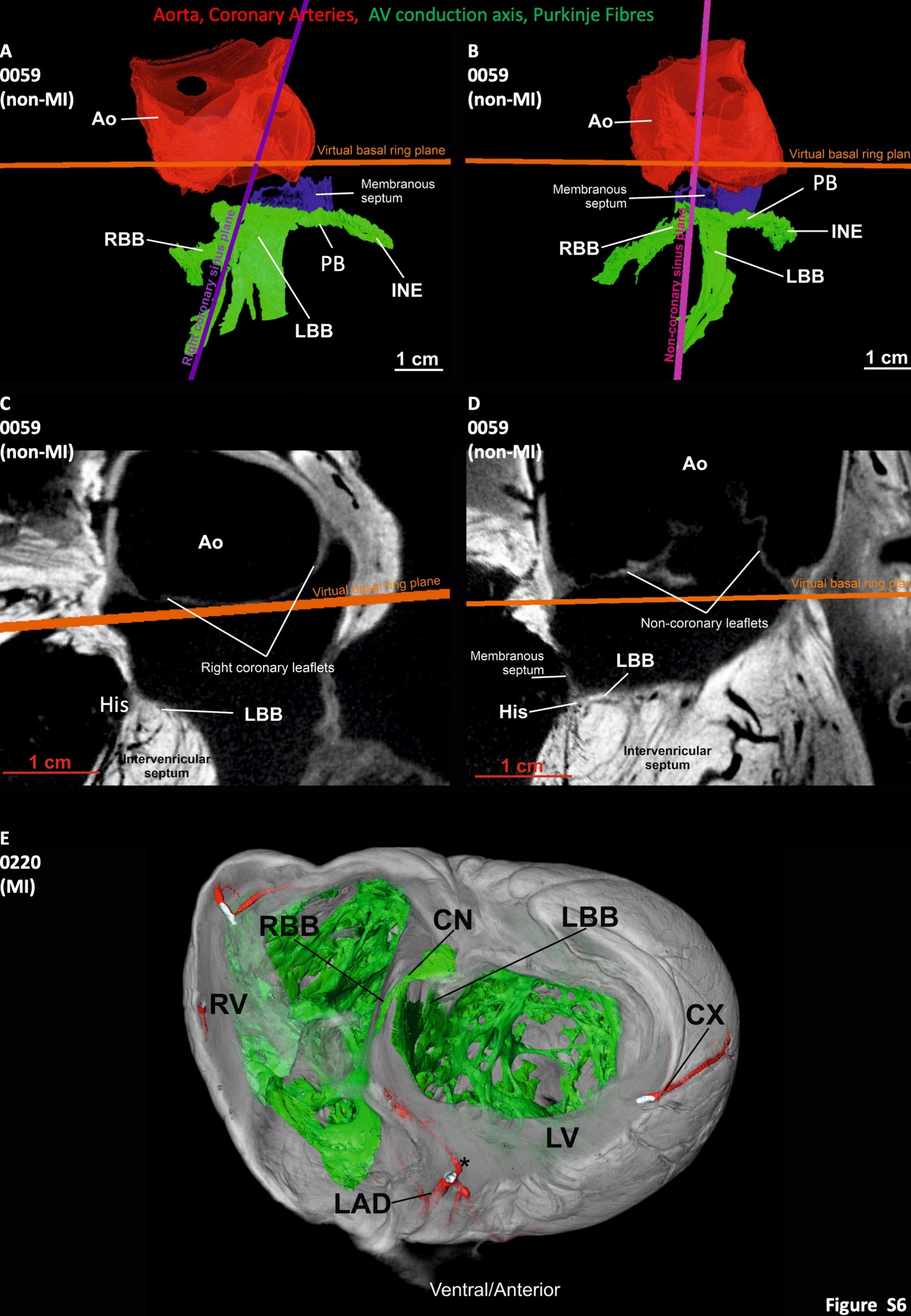

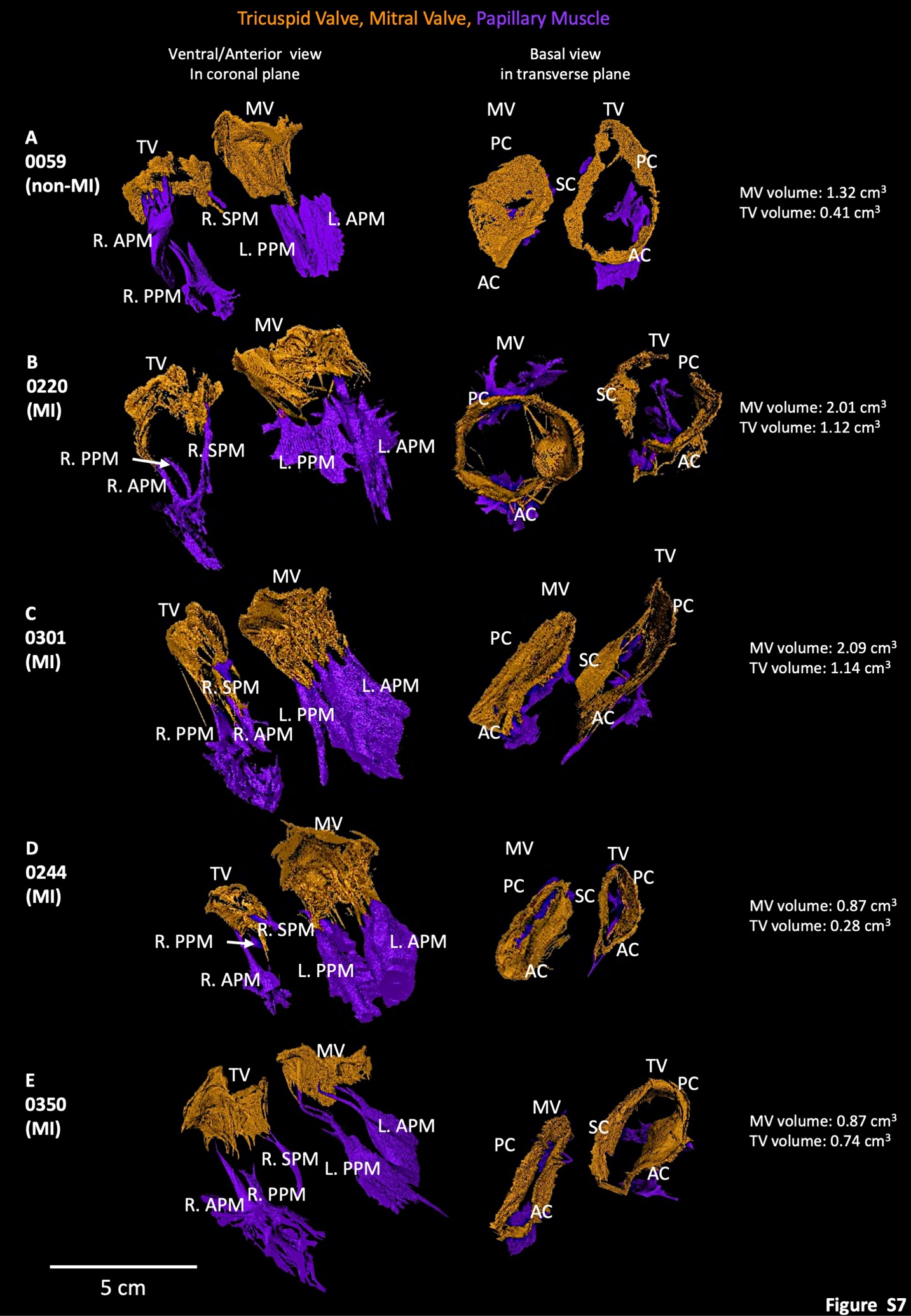

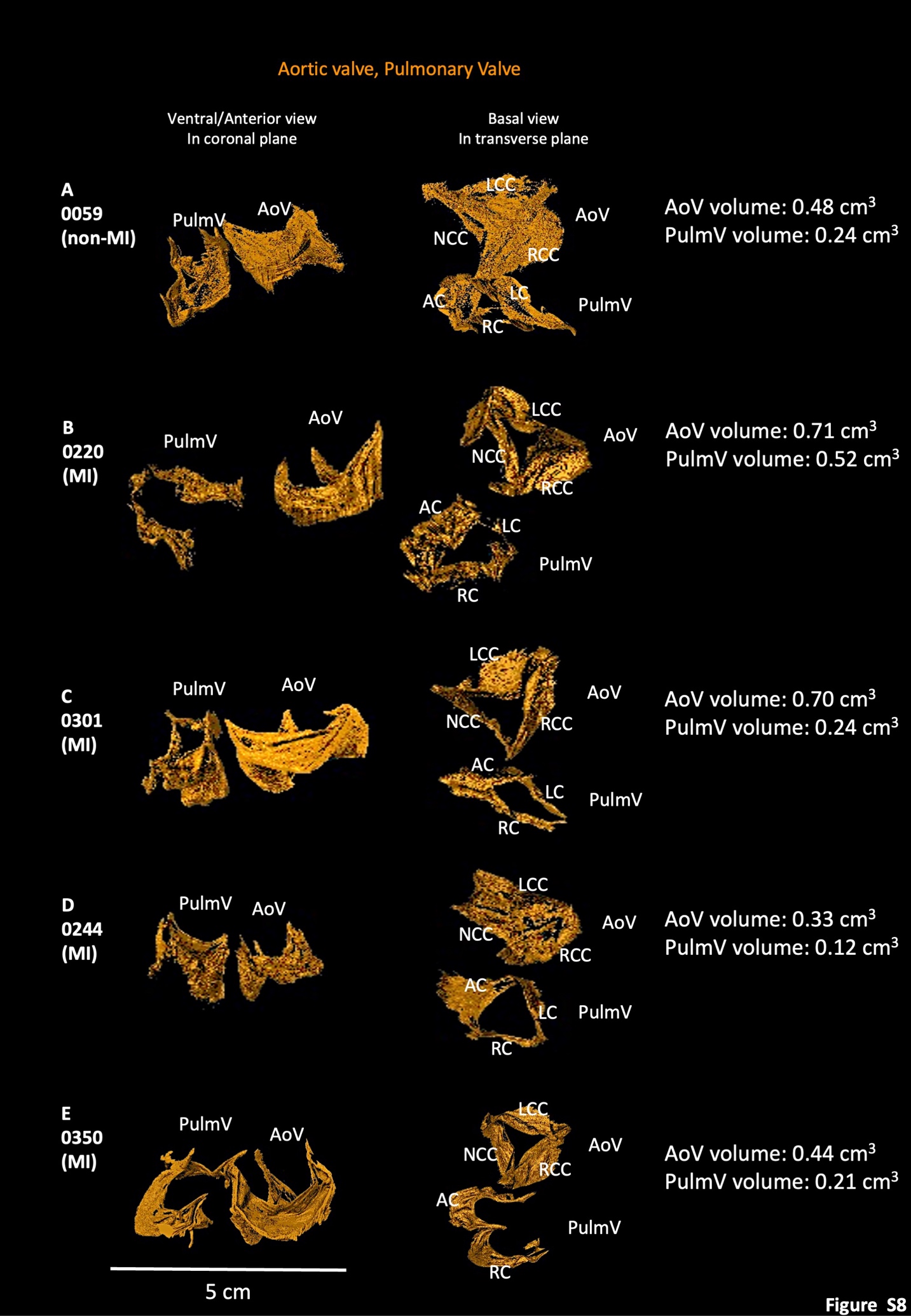

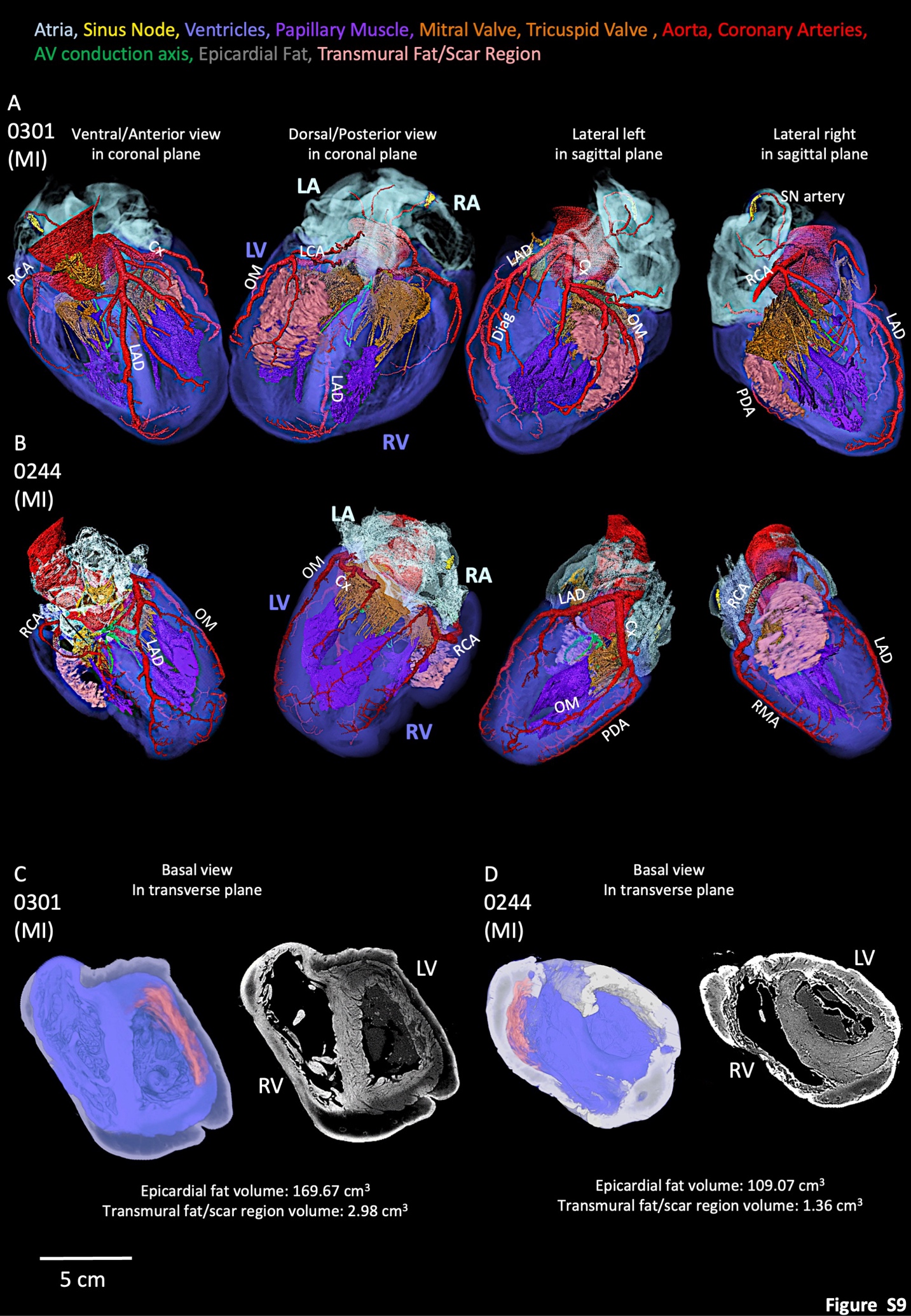

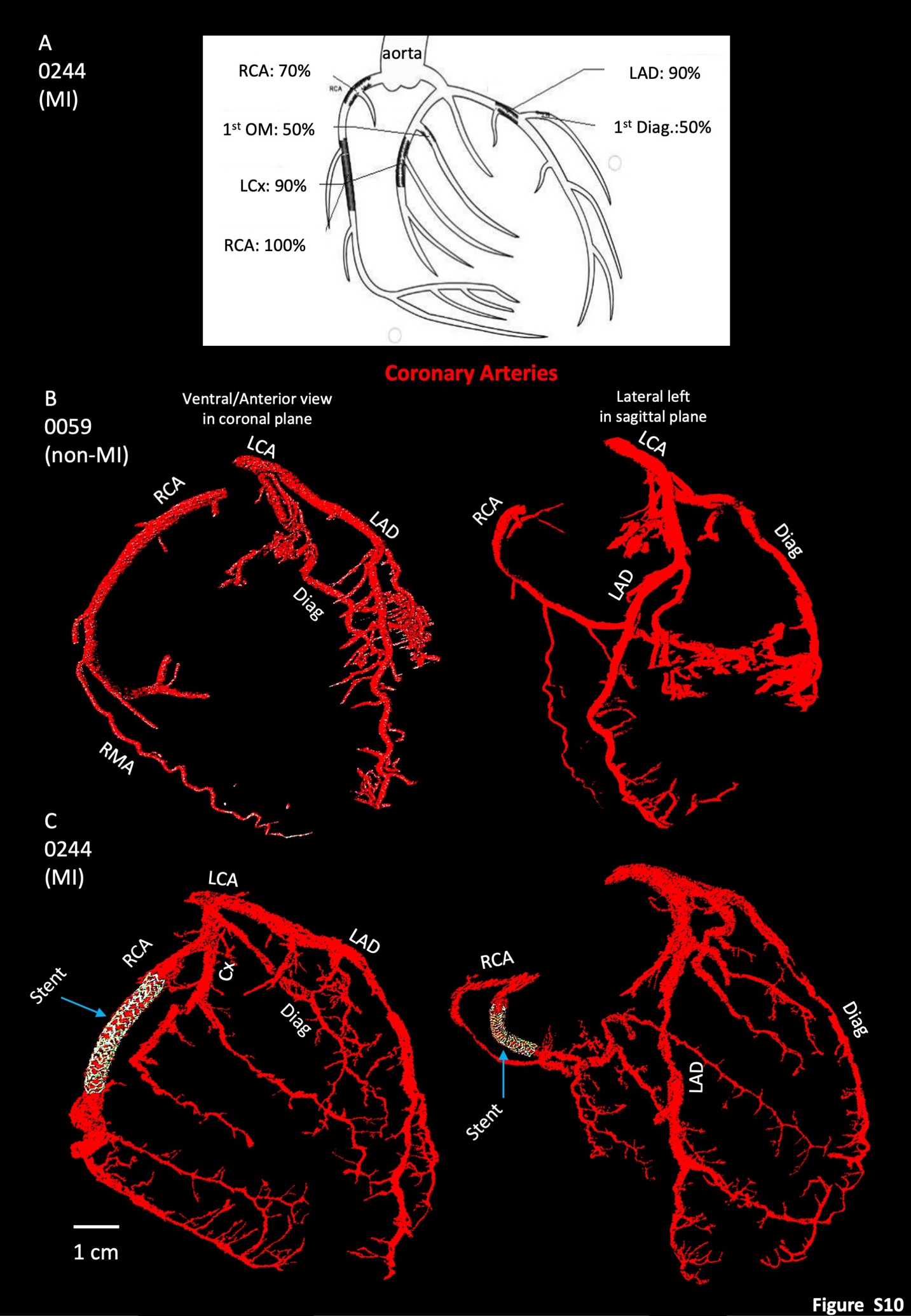

Supplement: Supplementary file 15 [file Data_Sheet_1.docx]
